# Supplementary material for: Implementation of Interventions and Policies on Opioids and Awareness of Opioid-Related Harms in Canada: A Multistage Mixed Methods Descriptive Study
Source: Int J Environ Res Public Health. 2022 Apr 22;19(9):5122. doi: 10.3390/ijerph19095122 (PMC9099533; doi:10.3390/ijerph19095122)

Supplementary material

Overview of literature search results (last updated on 15 November 2019)

Search Scope	Search Period	Bibliographical databases		Total abstracts *Including duplicates*
		MEDLINE	Embase	
Interventions (Risk minimization measure, risk minimization) for the opioid crisis in Canada	01 January 2016 – 15 November 2019	164	1,778	1,942

MEDLINE

#	Search terms	Results
**1**	Pharmacovigilance/ or Risk Management/ or Public Health/ or Health plan implementation/ or Preventive health services/ or Health Policy/ or Health Care Reform/ or Product Surveillance, Post marketing/ or risk minim*.ab,ti.	208,198
**2**	Analgesics, Opioid/ or morphine derivatives/ or codeine/ or dihydromorphine/ or ethylmorphine/ or heroin /or hydromorphone/ or morphine/ or oxymorphone/ or thebaine/ or hydrocodone/ or oxycodone/ or naloxone/ or naltrexone/ or buprenorphine/ or butorphanol/ or etorphine/ or levallorphan/ or levorphanol/ or loperamide/ or nalbuphine/ or nalorphine/ or papaverine/ or Opium/ or fentanyl/ or alfentanil/ or sufentanil/ or Methadone/ or Pentazocine/ or Tramadol/ or Phenoperidine/ or Pirinitramide/ or Dextropropoxyphene/ or Dextromoramide/ or Tapentadol/ or Meperidine/ or Tilidine/ or Cyclazocine/ or levopropoxyphene/ or Alphaprodine/ or (Opio?d*, Th?bacon* or Dipipanone or Ketobemidone or C?tob?midone or Profadol or Norm?thadone or Anil?ridine or Amidone* or Laudanum or Ac?tylm?thadol or Ac?tylcod?ine or Carfentanil or Ph?nazocine or M?tazocine or furanylfentanyl or ac?tylfentanyl or m?thoxyac?tylfentanyl or cyclopropylfentanyl or butyrylfentanyl or acrylfentanyl or m?thylfentanyl or norfentanyl or normorphine or normeperidine or U-47700 or MT-45).ab,ti.	131,112
**3**	1 and 2	802
**4**	limit 3 to (abstracts and humans and yr="2016 -Current")	164

Embase

#	Search terms	Results
**1**	preventive health service/ or risk management/ or "risk evaluation and mitigation strategy"/ or risk reduction/ or harm reduction/ or healthcare policy/ or public health/ or drug surveillance program/ or risk minim*.ab,ti.	522,265
**2**	opiate/ or naltrexone/ or narcotic agent/ or dextropropoxyphene/ or acetylcodeine/ or acetylmethadol/ or alphaprodine/ or anileridine/ or buprenorphine/ or codeine/ or dextromoramide/ or dihydromorphine/ or dipipanone/ or ethylmorphine/ or etorphine/ or hydrocodone/ or hydromorphone/ or ketobemidone/ or loperamide/ or levopropoxyphene/ or levorphanol/ or metazocine/ or methadone/ or morphine/ or morphinone/ or nalbuphine/ or normorphine/ or oxycodone/ or phenoperidine/ or piritramide/ or profadol/ or thebaine/ or tilidine/ or dextropropoxyphene/ or alfentanil/ or carfentanil/ or fentanyl/ or sufentanil/ or oxymorphone/ or morphine derivative/ or naloxone/ or levallorphan/ or diamorphine/ or tramadol/ or tapentadol/ or pentazocine/ or papaverine/ or butorphanol/ or phenazocine/ or cyclazocine/ or (furanylfentanyl or ac?tylfentanyl or U-47700 or MT-45 or th?bacon* or norm?thadone or m?p?ridine or norm?p?ridine or m?thylfentanyl or acrylfentanyl or butyrylfentanyl or cyclopropyl fentanyl or m?thoxyac?tylfentanyl).ab,ti.	331,521
**3**	1 and 2	8,266
**5**	limit 3 to (abstracts and human and yr="2016 -Current")	1,778

Table S1. Number of RMIs and policies implemented at national and provincial level between 2016 and 2019

Level of intervention implementation	n (%) (N=413)
Federal level	129 (30.9)
Provincial level	
Alberta	35 (8.4)
British Columbia	99 (23.7)
Manitoba	13 (3.1)
New Brunswick	2 (0.5)
Newfoundland and Labrador	13 (3.1)
Northwest Territories	1 (0.2)
Nova Scotia	4 (1.0)
Nunavut	2 (0.5)
Ontario	76 (18.2)
Prince Edward Island	1 (0.2)
Quebec	19 (4.6)
Saskatchewan	9 (2.2)
Yukon	5 (1.2)
Mixed	5 (1.2)

Table S2. Suspected-health products characteristics causing opioid-related harms in Canada Vigilance between 2009 and 2019

Suspected health products[a]	N(%)
**Opioid name (active ingredient)**	**(N=6,751)**
Oxycodone	2,047 (30.3)
Hydromorphone	1,402 (20.8)
Morphine	1,136 (16.8)
Fentanyl and its derivatives	693 (10.3)
Codeine	651 (9.6)
Methadone	229 (3.4)
Heroin	209 (3.1)
Buprenorphine	118 (1.7)
Pentazocine	87 (1.3)
Meperidine	60 (0.9)
Meperidine	60 (0.9)
Tramadol	55 (0.8)
Hydrocodone	45 (0.7)
Dextropropoxyphene	8 (0.1)
Oxymorphone	5 (0.1)
Opium	4 (0.1)
Normethadone	1 (0.01)
Levorphan	1 (0.01)
**Route of administration**	**(N=6,751)**
Oral	518 (7.7)
Transdermal	144 (2.1)
Intra-nasal	79 (1.2)
Transplacental	34 (0.5)
Inhalation	33 (0.5)
Sublingual	29 (0.4)
Subcutaneous	24 (0.4)
Intramuscular	20 (0.3)
Parenteral	17 (0.3)
Topical	6 (0.1)
Transmammary	4 (0.1)
Intradermal	4 (0.1)
Intrathecal	4 (0.1)
Intravenous drip	3 (0.04)
Buccal	3 (0.04)
Epidural	2 (0.03)

Rectal	2 (0.03)
Unknown	5,823 (86.3)
**Dosage form**	**(N=6,751)**
Short acting	3,638 (53.9)
Extended release	358 (5.3)
Long acting	124 (1.8)
Not specified	2,631 (39.0)

[a] Some case reports reported more than one opioid, thus more than one route of administration and dosage form.

Table S3. Suspected opioids causing deaths in Quebec (Coroner's reports) between 2009 and 2019

**Suspected opioid causing death**[a]	**N(%) (n=2,434)**
Hydromorphone	557 (22.9)
Morphine	425 (17.5)
Fentanyl and its derivatives[b]	323 (13.3)
Oxycodone	313 (12.9)
Methadone	219 (9.0)
Codeine	216 (8.9)
Heroin	216 (8.9)
Oxymorphone	65 (2.7)
Noroxycodone	25 (1.0)
Hydrocodone	23 (0.9)
Levorphanol	17 (0.7)
U-47700	14 (0.6)
Meperidine	8 (0.3)
Buprenorphine	6 (0.2)
Normeperidine	3 (0.1)
Norbuprenorphine	2 (0.08)
Norpropoxyphene	1 (0.04)
Unknown	1 (0.04)

Data are presented as n (%)

[a] Some deaths reports reported more than one opioid.

[b] Includes fentanyl, carfentanyl, norfentanyl, acetyl-fentanyl, furanyl fentanyl, fluorobutyryl fentanyl, par-fluorobutyryl fentanyl.

Figure S1. Reporting rate of opioid-related harm case reports (n=4,970) initially received in Canada Vigilance between 2009 and 2019

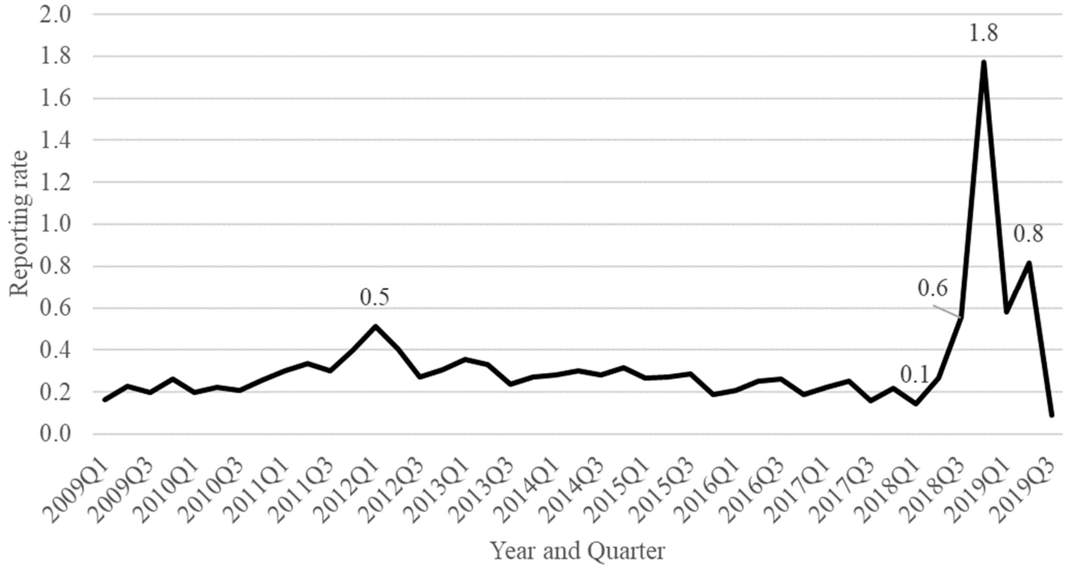

Denominator of the reporting rate is the size of Canadian population during the quarter (https://www150.statcan.gc.ca/t1/tbl1/en/tv.action?pid=1710000901&cubeTimeFrame.startMonth=01&cubeTimeFrame.startYear=2009&cubeTimeFrame.endMonth=07&cubeTimeFrame.endYear=2019&referencePeriods=20090101%2C20190701)

Figure S2. Number of opioid-related harm case reports according to the type of reporter between 2009 and 2019 in Canada Vigilance

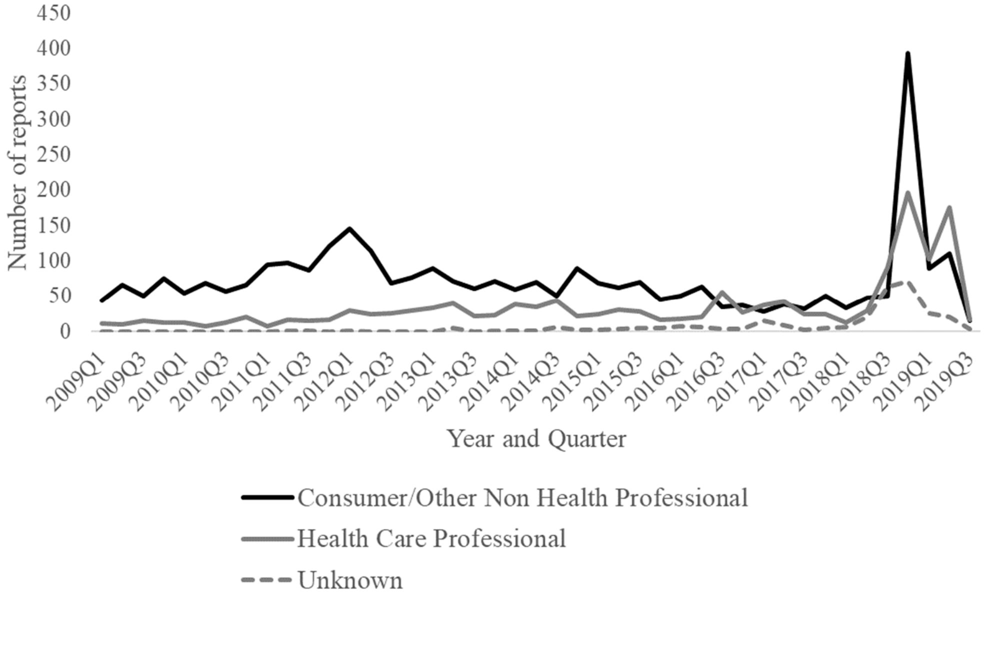

Figure S3. Number of opioid-related deaths in Quebec caused by fentanyl and its derivatives (n=271) and hydromorphone (n=448) between 2009 and 2019

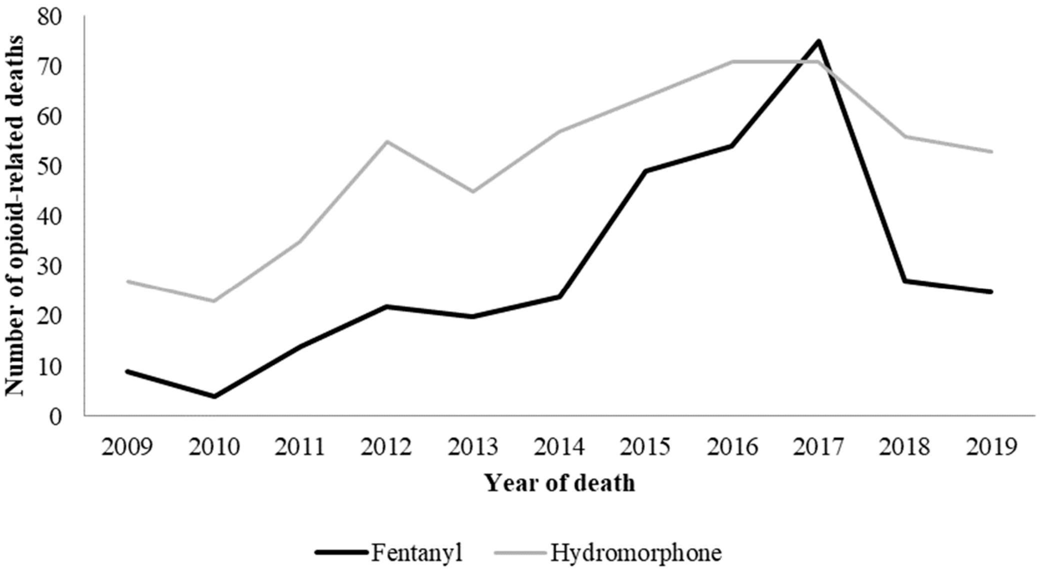

Figure S4. Relative distribution of fentanyl-, hydromorphone-, and oxycodone-related deaths in Quebec between 2009 and 2019

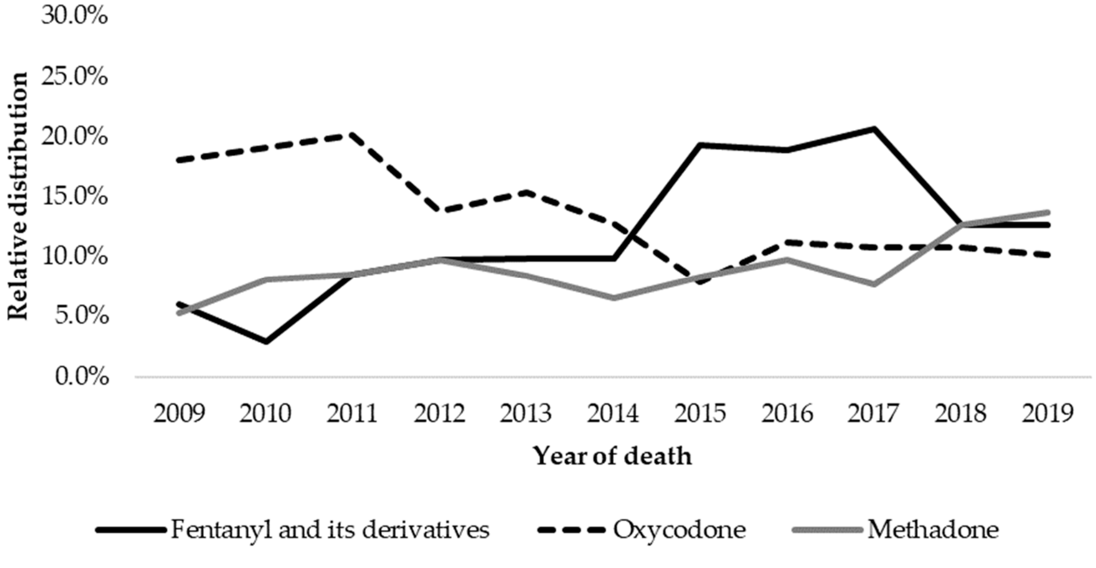

Supplement: Supplementary file 1 [file ijerph-19-05122-s001.zip › ijerph-1617809-supplementary.pdf]
